# Achieving Salt Restriction in Chronic Kidney Disease

**DOI:** 10.1155/2012/720429

**Published:** 2012-12-23

**Authors:** Emma J. McMahon, Katrina L. Campbell, David W. Mudge, Judith D. Bauer

**Affiliations:** ^1^Nutrition and Dietetics Department, Princess Alexandra Hospital, 199 Ipswich Road, Woolloongabba, Brisbane, QLD 4102, Australia; ^2^School of Human Movement Studies, University of Queensland, Blair Drive, St Lucia, Brisbane, QLD 4072, Australia; ^3^Department of Nephrology, Princess Alexandra Hospital, University of Queensland, Ipswich Road, Woolloongabba, Brisbane, QLD 4102, Australia

## Abstract

There is consistent evidence linking excessive dietary sodium intake to risk factors for cardiovascular disease and chronic kidney disease (CKD) progression in CKD patients; however, additional research is needed. In research trials and clinical practice, implementing and monitoring sodium intake present significant challenges. Epidemiological studies have shown that sodium intake remains high, and intervention studies have reported varied success with participant adherence to a sodium-restricted diet. Examining barriers to sodium restriction, as well as factors that predict adherence to a low sodium diet, can aid researchers and clinicians in implementing a sodium-restricted diet. In this paper, we critically review methods for measuring sodium intake with a specific focus on CKD patients, appraise dietary adherence, and factors that have optimized sodium restriction in key research trials and discuss barriers to sodium restriction and factors that must be considered when recommending a sodium-restricted diet.

## 1. Introduction

Reducing cardiovascular risk and chronic kidney disease (CKD) progression are two of the primary goals of CKD management [1, 2]. Excessive sodium intake has been extensively researched for its relationship with cardiovascular risk factors, in particular hypertension and volume overload [3–5]. New research has indicated that dietary sodium intake may influence other novel, uremia-related risk factors in CKD, including oxidative stress, proteinuria, inflammation, and endothelial cell damage, and can increase cardiovascular risk, independent of blood pressure changes [2, 6–9].

Individuals with impaired kidney function may be particularly susceptible to the adverse effects of excessive sodium intake [2]. As sodium handling is primarily the role of the kidney [10], those with CKD may have a reduced ability to excrete sodium, making them less able to compensate for the high sodium load that is characteristic of the Western diet [10–12]. A recent review of the evidence for the relationship between salt intake and CKD progression concluded there is consistent evidence to suggest that dietary salt intake is linked with albuminuria and tissue injury, although acknowledged that empirical evidence is lacking [7]. Increased sodium intake is linked to reduced GFR [6], although this is somewhat controversial; increased sodium intake leads to increased glomerular pressure [13] causing short-term hyperfiltration that initially increases GFR [14]. However, this increased glomerular capillary pressure increases proteinuria, an independent risk factor for GFR decline and CVD [15, 16]. Several trials have indicated improved efficacy of antiproteinuric agents with decreased sodium intake, suggesting that sodium can act as an antagonist to these drugs [6, 17]. Animal studies have demonstrated increased proteinuria on a high sodium compared to a low sodium diet [18] and a relationship between sodium intake and albuminuria has been observed in the general population [16], although this has not been well explored in CKD.

There is compelling evidence that reducing dietary sodium can reduce cardiovascular risk and risk for kidney function decline in CKD patients, while being a cost-effective intervention with low risk of adverse effects [19]. However, like medications will only be effective if taken as prescribed, a recommendation to reduce dietary sodium will only reduce cardiovascular and CKD progression if adherence is achieved. Poor adherence to sodium restriction is an ongoing problem in research trials and in the clinical practice setting [20]. Nonadherence in these two settings present two distinct issues: nonadherence in research trials may greatly underestimate the effect of the intervention, obscuring the diet-disease relationship, while nonadherence in the practice setting can impede reduction of disease risk, as well as interfering with the efficacy of some medications, potentially increasing risk of adverse outcome. The aim of this critical paper is to evaluate adherence to sodium restriction, including measurement methods, the degree of adherence achieved in research trials, recommendations for intake, and factors related to greater adherence to sodium restriction.

## 2. Measurement of Sodium Intake in CKD

It is imperative to measure dietary adherence in a dietary intervention trial to ensure changes seen in outcomes are attributable to changes in intake. There are several objectives and self-reported methods available for measurement of sodium intake; however, there are errors inherent in all methods and the strengths and limitations of each method must be taken into consideration (see Table 1) [21].

### 2.1. Urinary Sodium

As nearly all sodium ingested is excreted in the urine, repeated 24-hour urine measurements are considered by the World Health Organisation to be gold standard [21]. However, as sodium intake can vary significantly from day to day, accuracy of 24-hour urinary excretion to reflect sodium intake over a given time is directly related to the number of collections gathered [22]. When examining the potential effect of day to day variation in sodium intake on outcomes in research trials, Lui et al. estimated that the correlation between sodium and an outcome variable (e.g., blood pressure) could be weakened by half if a single measurement of 24-hour urinary sodium excretion was used [23]. This study estimated that even with four measurements of 24-hour urinary sodium excretion, potential correlations could be diminished by 25% (compared with 10 days) [23]. However, increased number of samples involve higher participant burden, increasing the likelihood of error related to improper collection [21]. For this reason, it is recommended to supplement 24-hour urinary sodium measurements with other methods to maximise accuracy of estimated dietary sodium intake [21].

Spot urinary sodium, or sodium to creatinine ratio (Na : Cr), is an objective measure of dietary sodium intake with relatively smaller participant burden than 24-hour collection. The validity of this measure to represent sodium intake is contentious, particularly in CKD where excretion of these solutes may be deranged [12]. There has been little research in this area; however, two recent studies provide favourable results as a surrogate marker of sodium intake [24, 25]. Ogura et al. (2012) [26] assessed spot urinary sodium values extrapolated to estimations of 24-hour urinary sodium excretion using Tanaka's formula [26] and the agreement with actual 24-hour sodium excretion in 96 CKD patients [25]. Using Bland-Altman analysis, mean difference between estimated (from spot urine) and measured 24-hour urinary sodium excretion was −10.9 mmol with 94% of the values lying within 1.96 standard deviations of the mean difference, suggesting good agreement [25]. Kang et al. also compared spot urinary sodium to 24-hour urinary sodium excretion in 305 CKD patients, and found that the correlation between mean of three spot samples taken at different times of the day and 24-hour urinary sodium excretion was 0.477 (95% CI: 0.384–0.562; *P* < 0.01) for spot urinary sodium and 0.313 (0.207–0.415, <0.01) for spot urinary sodium to creatinine ratio [24]. However, these correlations, although significant, are weak and indicate associations rather than agreement. Given the lower participant burden and that spot urinary samples are often taken as part of standard practice, the validity of spot sodium or Na : Cr to represent intake in CKD warrants further investigation.

### 2.2. Dietary Assessment Methods

Taking into consideration the limitations of urinary sodium measures when estimating habitual intake, it is wise to employ self-reported dietary methods alongside these objective methods [21, 27]. There are several methods of self-report available to assess sodium intake; the method chosen should consider the inherent strengths and limitations (see Table 1), as well as the aim of the study, the population in question and time and resources available. Dietary intake assessment methods have been comprehensively reviewed (with respect to the general population) elsewhere [21]. As the validity of a self-reported measure of sodium intake is unlikely to differ when used in those with or without impaired kidney function, this paper will discuss self-reported methods only briefly.

The diet history, where information about what is eaten over an extended period of time is collected using open-ended questions [28], is considered to be a useful method for capturing usual intake [29] and aligns closest to dietary assessment methods used in clinical practice [30]. This method has performed favourably when compared with other dietary assessment methods such as food records or 24-hour recall [31] and has been comprehensively reviewed with respect to nutrients other than sodium [29]. A study of 60 young omnivores and vegans in Sweden found sodium estimations from diet history to be within 9 mmol (198 mg) of mean urinary sodium excretion from four 24-hour urine collections [32], suggesting good agreement. There are several advantages inherent in the diet history method; the presence of interviewer maintains participant interest and enables clarification of misunderstandings, information collected is not limited to a finite list of foods, and an interviewer skilled in dietary methodology is able to target foods that are likely to contribute significant amounts of sodium to the diet [29, 33]. However, this method, like all retrospective methods (i.e., diet history, 24-hour recall, food frequency questionnaire), is susceptible to error related to memory lapse and reporting bias [34]. In addition, accuracy of the method depends on the skills and training of the interviewer [27, 30].

Food records (or food diaries) are a common method for measuring intake in dietary trials as they are easy to administer and the prospective nature of the method limits error related to memory lapse [29]; however, the accuracy of this method to assess usual intake is questionable. Systematic bias is found, with a tendency to underestimate intake compared with objective markers [35–37]. For example, Day et al. found that a 7-day food record underestimated intake by approximately 17% when compared with urinary sodium excretion [35]. Correct recording of actual intake requires motivated participants [38]. In addition, the burden of keeping accurate food records can affect intake; study participants have reported reduced intake of snacks and other foods, and decreased complexity of the diet on days that food records are kept [39, 40]. For example, in the Hypertension Prevention Trial, 48% of participants reported modifying their diet on days where records were kept, with those who were assigned a sodium-restricted diet eating more low sodium foods (or less salt/salty foods) on recording days [36]. While increasing the number of recording days increases likelihood of capturing “usual intake,” this also increases participant burden. Rebro et al. found intake calculated from food records was lower on day four compared with day one, with lower number of foods recorded, and lower estimates of energy, carbohydrate, and other nutrients [39]. For these reasons, using food records to indicate intake over an entire intervention, or “usual” intake in a practical setting, may misrepresent intake [41].

The 24 hr recall method refers to collecting information retrospectively about all foods and fluids consumed over the previous 24 hours [29]. This method is quick and inexpensive to administer, but does not account for day-to-day variation in intake [42, 43], meaning that it may not give an accurate estimation of habitual intake, unless repeated [44]. This method is also subject to memory lapse and reporting bias. When 24-hour recall was compared to 24-hour urinary collection, Espeland et al. found that participants underestimated their sodium intake by 22% and that the underestimation was larger in the treatment group recommended low sodium than other groups [45, 46]. In order to decrease error by memory lapse, the United States Department of Agriculture (USDA) developed the five-step multiple-pass 24-hour recall method which consists of steps where participants: list the foods, are questioned on categories of foods that have been documented as frequently forgotten, report the time and occasion foods were consumed, are asked for further information on descriptions of foods and amounts eaten (aided by the use of the USDA Food Model Booklet and measuring guides), and finally review of information given [47]. Validation studies specific to sodium are lacking, but when validated against estimates of energy intake from doubly labelled water [48] and direct observation [49], this method has been found to be a valid tool for estimation of group intake, but not to give a precise estimate of individual intake, suggesting it may be most suited to epidemiological research.

A food frequency questionnaire (FFQ) is a questionnaire in which the respondent is presented with a list of foods and is asked to identify the frequency each is eaten in broad terms (e.g., per day/per week) [50]. Charlton et al. developed a FFQ specifically to estimate sodium intake and found that the estimation of sodium was strongly correlated with repeated 24-hour sodium excretion (*r* = 50.683, *P* < 0.0001) [51]. However, the FFQ had a sensitivity of only 12.4% (27/218) to classify patients as having an intake under <100 mmol and specificity of 93.9% (62/66) to identify patients with intake ≥100 mmol suggesting the questionnaire may underestimate intake. Other studies that have validated FFQs—designed to quantify a range of nutrients including sodium—against urinary sodium excretion have reported similar results [35, 52]. One of the difficulties with using a FFQ to estimate sodium intake stems from the ubiquitous nature of sodium in the food supply, meaning that a comprehensive list of foods is needed to capture intake. In addition, memory lapse, reporting bias, difficulty estimating portion sizes, and difficulty calculating discretionary salt usage can further increase risk of error [51, 53, 54]. Furthermore, differences in food supply and intake between populations may mean a particular FFQ and its scoring may be more accurate in some populations than others [55].

## 3. Adherence to Sodium Restriction in Clinical Trials

Several studies of sodium restriction have experienced difficulty with patient adherence to the specified sodium target [8, 56, 57].  Table 2 shows a summary of adherence in key trials of sodium restriction. Notably, those with intensive methods for delivering the interventions, particularly those that employed total food provision, achieved excellent adherence [4, 58]. The DASH trial employed several methods to ensure dietary adherence [58]. All food was provided to participants for the month long study, a minimum one meal per day was eaten on-site (decreasing opportunities for nonadherence), and incentives including prizes and monetary reimbursement were provided to encourage adherence [58]. Multiple methods to measure dietary adherence were also used, including 24-hour urinary sodium excretion, daily diaries and a poststudy anonymous survey. These parameters indicated excellent adherence, with a mean urinary sodium excretion of 67 ± 46 and 64 ± 37 mmol (DASH and control groups) for the strictest dietary sodium target of 50 mmol per day, and complementary methods (daily diaries, poststudy survey) confirming excellent adherence. However, large standard deviations for urinary sodium excretions in the low sodium groups indicate considerable variability in adherence between participants [58].

Pimenta et al. used total food provision as a means for delivering the low sodium intervention and achieved a mean urinary sodium excretion of 46 ± 27 mmol per day (target 50 mmol/day) [4], indicating closer adherence than that achieved in DASH with smaller variation between participants, although the intervention duration was considerably shorter. While these studies provide an indication of efficacy of sodium restriction for reducing BP in their respective populations, intensive methods such as these require significant monetary and other resources (cooking facilities, cafeteria for dining in) which are not always available to researchers or feasible for participants. In addition, they do not provide any indication of the effectiveness (i.e., whether these results are achievable in a practical setting). 

Troyer et al. investigated whether providing only one meal per day to elderly participants for one year improved adherence to sodium restriction [59]. The dietary intake data shows negligible change in sodium intake at 6 or 12 months from baseline in both groups; however, this may be a reflection of the method for gathering dietary intake data, given that as 24-hour recall does not assess daily deviations in intake and may be subject to memory lapse. 

Examples of trials where excellent dietary adherence was achieved without provision of food are provided by Luik et al., Gates et al., and Todd et al., where dietary education was used as the primary means for delivering the interventions (see Table 2) [14, 60, 61]. A key characteristic common to these studies is that the dietary education was individualized, often employing the use of registered dietitians, who have thorough knowledge of the nutritional content of foods as well as extensive training in dietary data collection and experience implementing dietary interventions. As this more closely reflects what occurs in usual practice when a patient is prescribed a low sodium diet, these trials can be said to measure effectiveness as well as efficacy of sodium restriction in their populations. 

The literature provides numerous examples of sodium restriction trials that rely on single 24-hour urinary sodium collection to indicate dietary intake during the entire intervention [62, 63]. However, doing so assumes that this single 24-hour collection is representative of sodium intake over the entire intervention, and, as described previously, using a single measurement can considerably obscure the diet-disease relationship [23].

## 4. Sodium Intakes in the Clinical Setting

Most guidelines specific to the nutritional management of CKD patients recommend an upper limit of 100 mmol of sodium per day (2300 mg, 6 g NaCl) [66–69]. The United States dietary guidelines recommend a stricter target of <65 mmol per day for people with CKD (1495 mg, 3.8 g) [70]. Mean 24-hour urinary sodium excretion in large studies suggests that CKD patients commonly have urinary sodium excretions between 150 and 200 mmol, far above the recommendations [71–75]. In fact, the rate of adherence to a sodium target of less than 100 mmol per day is met by only 13–19% [2, 75, 76]. 

Influences on dietary behaviors are complex and mediated by a number of factors [71]. Barriers specific to dietary sodium restriction that arise frequently in the literature can be summarized as being related to

- perceived taste/palatability of low sodium foods,
- convenience/difficulty (e.g., time, availability of low sodium foods, interference with socialization, and cost) or,
- lack of knowledge or understanding (e.g., lack of perceived benefit and inability to identify low sodium foods) (Table 3 [20, 65, 77–79]).

An observational study by Welch et al. measured barriers and enablers to following a low sodium diet in hemodialysis patients in the USA [20]. While nearly all participants indicated that they agreed that a low salt diet would make them feel better and keep their blood pressure down, the majority reported taste as a barrier. In addition, a considerable proportion of participants reported difficulty when eating out, cost, and difficulty understanding a low sodium diet as barriers [20].

De Brito-Ashurst et al. also examined barriers to a low sodium diet in immigrants to the UK and emphasized the importance for individualized messages, stating that “provision of generic low sodium information had led participants to believe they did not have a high sodium intake, paradoxically deterring dietary sodium reduction” [72]. Health care practitioners may find it is common for patients to report that they consume very little salt, despite having high urinary sodium excretion. Discretionary salt (salt added to food) is a relatively small contributor to sodium intake compared with salt already in the foods on the shelves [73, 74]. There are many foods that do not taste salty despite high sodium content, for example, certain breakfast cereals and sweet biscuits, making it difficult to follow a low sodium diet without paying close attention to nutrition information panels.

Evidence suggests that hedonic liking and taste sensitivity of salty taste can be modified with adherence to a low sodium diet [75]. Mattes studied 8 normotensive adults and, while at baseline regular sodium products were preferred to reduced sodium versions, after four months of sodium restriction there was no significant difference in preference for these products [76]. Kusaba et al. found that taste sensitivity in CKD patients was improved after only one week of adherence to a low sodium diet [62]; however, given the short-term nature of the study, and the fact that adherence to the sodium-restricted diet was not measured (although all meals were consumed on site as participants were inpatients), this is an area that warrants further investigation.

## 5. Conclusion and Recommendations

Excessive sodium intake shows promise as a modifiable risk factor to reduce cardiovascular risk and risk for CKD progression in CKD patients; however, research in this area is not yet conclusive. Researchers planning to explore this area further must take the issues presented in this paper into consideration; it is imperative to measure sodium intake in a sodium restriction trial (or any trial where sodium is a variable that could affect the outcome in question). The measurement methods chosen to assess sodium intake must consider the inherent limitations. Undertaking a panel of measures is optimal. 

Using a single 24-hour urinary sodium excretion to represent intake over a dietary intervention can greatly underestimate the efficacy of sodium reduction. While repeated 24-hour sodium excretion is gold standard for measuring intake, these are not always practical. Spot urinary sodium samples entail considerably lower participant burden, but further research in CKD needs to be conducted to ascertain if these are valid indicator of total intake. Supplementing 24-hour urine samples with other self-reported measures such as open-ended, interviewer-administered diet history is recommended.

Patient adherence to sodium restriction must be taken into account when examining the effect on a particular outcome. Several trials have reported poor adherence. Tightly controlled feeding studies have achieved excellence adherence, but researchers do not always have the resources required to conduct these. Where dietary education is given, using an individualized approach may enhance adherence.

Sodium intakes in the general population and in CKD patients are far above that recommended. Health care practitioners must take barriers to sodium restriction into consideration when recommending a low-sodium diet. Using an individualized approach is also useful in practice.

### 5.1. Key Recommendations

- When designing a research trial where sodium intake is to be measured, consider the strengths and limitations of available methods.
- Where possible, use a panel of methods, including self-report (e.g., diet history method) alongside objective markers (e.g., 24-hour urinary sodium) to assess intake.
- Food provision and provision of individualized dietary education aid adherence to sodium restriction in a research trial.
- Employing a registered dietitian skilled in dietary assessment and individualized dietary education may enhance the effectiveness of an intervention.
- Consider barriers to a low sodium diet when advising a low sodium diet; perceived taste/palatability of low sodium foods, convenience/difficulty, and lack of knowledge or understanding (e.g., lack of perceived benefit, inability to identify low sodium foods) are barriers to sodium restriction.

## Figures and Tables

**Table 1:** Summary of measures that can be used to estimate sodium intake.

Measure	Objectivity	Burden	Strengths	Limitations
24-hour urinary sodium	Objective	High	(i) Gold standard (ii) Accurately quantifies actual intake when collected correctly	(i) Under/overcollection can introduce error (ii) High participant burden (iii) High cost of analysis (iv) Does not account for daily variation

Spot urinary sodium	Objective	Low	(i) Low participant burden (ii) Quick to collect (iii) Can be used as part of standard practice	(i) Does not account for diurnal variation (ii) Further research needed to determine if valid indicator of daily intake

Open-ended diet history	Self-report	Moderate	(i) Most comprehensive of self-reported measures (ii) Presence of interviewer allows for verification and clarification of information provided (iii) Retrospective nature allows for assessment of “usual intake”	(i) Time consuming to collect and code (ii) Prone to memory lapse and reporting bias (iii) Specialized skills and knowledge of food supply required

24-hour recall	Self-report	Low	(i) Standardized (ii) Can be implemented over the phone (iii) Multiple administrations increases validity (iv) Best for larger population-based studies	(i) Does not account for daily variation in intake (unless repeated) (ii) Prone to reporting bias

Food records (diaries)	Self-report	Depends on number of days	(i) Precise estimation of actual intake *on the days where recording occurs*, when completed correctly	(i) Subject to participant motivation (ii) Increased number of days increases participant burden (iii) Prone to under-reporting (iv) Prone to modification of intake on days where recording occurs

Food frequency questionnaire	Self-report	Low	(i) Standardized (ii) Quick to administer (iii) Easily coded (iv) Many validation studies	(i) Validity can depend on population in question and food supply. (ii) Subject to memory lapse; inability to summarize intake; false perception of own intake. (iii) Collects information about a limited number of foods (iv) Difficult to measure discretionary salt usage

**Table 2:** Summary of adherence in key trials of sodium restriction.

Study	Population and design	Duration	Food provision	Dietary education	Measurement methods	Feedback	Sodium goal (mmol)	Actual intake (mmol)
Pimenta et al. (2009) [4]	*n* = 12 RCT-crossover	1 wk	Full	Nil	24 hr UNa (once), direct observation	Nil	50	46 + 27

Luik et al. (2002) [14]	*n* = 38 RCT-crossover	1 wk	Nil	Partial, individualized, at baseline only	24 hr UNa (once)	NS	50	Diabetics = 38 ± 13; Healthy = 45 ± 28

DASH-Sodium Sacks et al. (2001) [64]	*n* = 375 RCT-crossover	4 wk	Full	Partial, individualized with follow-up, frequency NS	24 hr UNa, daily diary, meal attendance, poststudy anonymous survey, plus monetary, and other incentives	Daily deviation record and met with dn	Low 50 and Mod 100	Low: 67 ± 46, Mod: 107 ± 52

Todd et al. (2010) [61]	*n* = 24 RCT-crossover	4 wk	Nil	Weekly, individualized, follow-up NS	3d FR, recall, UNa: Creatinine (after overnight fast)	24 hr recalls, met with dn (frequency NS)	60	78 ± 18

Gates et al. (2004) [60]	*n* = 12 RCT-crossover	4 wk	Nil	Weekly, individualized	3d FR; 24 hr UNa (weekly)	Met with dietitian	60	52 ± 4

Vogt et al. (2008) [8]	*n* = 33 RCT-crossover	6 wk	Unclear	Partial, individualized, follow-up NS	24 hr UNa (every 2 wk)	Unclear	50	90 ± 10

Ireland et al. (2010) [65]	*n* = 33 Randomized uncontrolled trial	8 wk	Nil	Group “Tick” versus Panel, not individualized, at baseline and wk 4	24 hr UNa, multiple pass 24 hr recall (at wk 4 and 8)	Unclear	85	Tick = 106 ± 47 FZANZ = 98 ± 50

Troyer et al. (2010) [59]	*n* = 210 RCT-parallel	52 wk	Partial (1 group)	Partial, unclear if individualized, follow-up NS	24 hr recall and 12 item FFQ	Nil	<50/1000 kcal	Meals = 65 ± 24 Non = 67 ± 28 mmol/kcal

Sodium as mmol/day unless otherwise specified. Abbreviations: d: day, dn: dietitian, FFQ: food frequency questionnaire, FR: food records, hr: hour, mod: moderate, *n*: sample size, Na: sodium, NS: not specified, RCT: randomized controlled trial, U: urinary, wk: week.

**Table 3:** Summary of studies investigating barriers to adhering to a sodium-restricted diet.

Study country	Population	Barriers to sodium-restricted diet
Welch et al. (2006) [20] USA	229 *hemodialysis* pts, aged 55 ± 14 years. 58% male, 79% African American	(i) Taste (58%) (ii) Difficulty when eating out (30%) (iii) Cost (23%) (iv) Difficult to understand (21%) (v) Too time-consuming (17%)

De Brito-Ashurst et al. (2011) [72] UK	20 female *CKD pts*, 1st generation immigrants from Bangladesh to the UK, aged 60 ± 8 years; unemployed	(i) Lack of family acceptance (50%, *n* = 10/20) (ii) Fear that friends will gossip/think the family has no money (40%, 8/20) (iii) No perceived benefit (25%, *n* = 5/20)

Gordon et al. (2009) [77] USA	82 *transplant recipients* aged 47 ± 57 years. 57% male, 56% white	(i) Preferences for salty foods and enjoying taste of salt (*n* = 9) (ii) Lack of available low-salt dishes at restaurants (*n* = 10) or low-salt foods in markets (*n* = 3) and when other people cook using salt (*n* = 3) (iii) Lifestyle factors (*n* = 5) for example, having no time to cook

Ireland et al. (2010) [65] Australia	43 *healthy pts* from volunteer database. 23% male, aged 55 ± 11 in “tick group” 57 ± 13 y in “FSANZ group”	(i) Limited variety of appropriate foods (ii) Difficulty eating out (iii) Increased time for shopping

Chung et al. (2006) [78] Australia and United States	68 *heart failure* patients, aged 63 ± 14 years, 60% male, 63% Caucasian	(i) Trouble choosing foods in restaurants (75%) (ii) Favorite foods aren't low-salt (72%) (iii) Taste (69%) (iv) Favorite restaurants don't serve low-salt foods (64%) (v) Insufficient will power to change diet (59%) (vi) Peers don't eat low-salt foods (54%) (vii) Trouble choosing foods at supermarket (52%) (viii) Poor knowledge/understanding (49%) (ix) Cost (47%) (x) Does not cook (40%) (xi) Time to prepare food (38%) (xii) Person who cooks doesn't prepare low-salt foods (30%)

Bentley et al. (2005) [79] USA	20 heart failure patients (recruited from 1 clinic) who had received a healthcare provider's recommendation to follow a low sodium diet, aged 60 ± 11 years 60% male, 80% non-Hispanic White	(i) Lack of knowledge (need for more detailed dietary information, confusion for pts with additional dietary restrictions) (ii) Lack of perceived benefit (iii) Interference with socialization (family conflict, difficulty eating out) (iv) Limited food choices/lack of palatability

Figures as mean ± standard deviation. Abbreviations: CKD: chronic kidney disease, FSANZ: Food Standards Australia New Zealand, Pts: participants.
